# Supplementary material for: Conditional chemoconnectomics (cCCTomics) as a strategy for efficient and conditional targeting of chemical transmission
Source: eLife. 2024 Apr 30;12:RP91927. doi: 10.7554/eLife.91927 (PMC11060718; doi:10.7554/eLife.91927)
Supplement: Figure 3—source data 3. [file elife-91927-fig3-data3.zip › Figure 3 source data3 part 2.zip!/Figure 3-Source data 3-part2/1053-1-CRISPResso_Report_mpeqg9da.zip!/CRISPResso_on_1053_1/3b.Insertion_deletion_substitutions_size_hist.pdf]

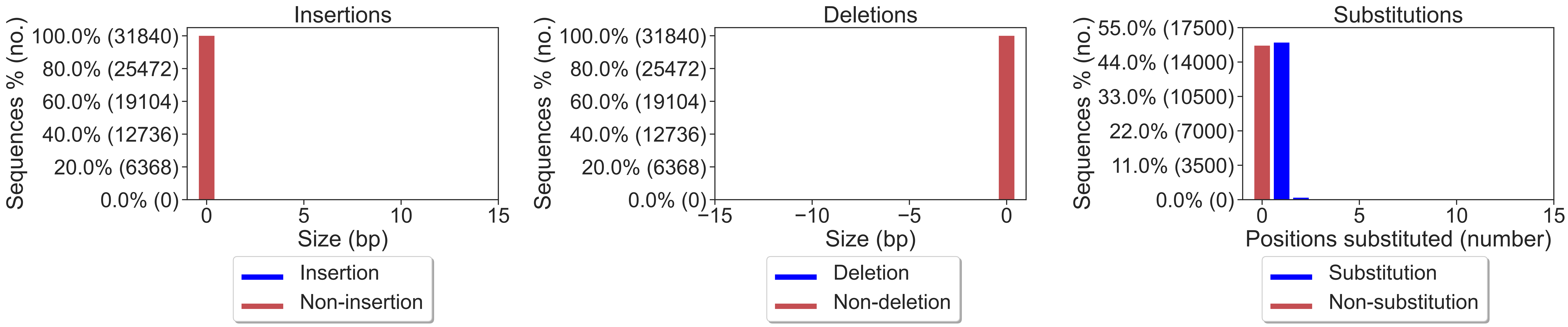
Insertions
Sequences % (no.)
100.0% (31840)
80.0% (25472)
60.0% (19104)
40.0% (12736)
20.0% (6368)
0.0% (0)
0
5
10
15
Size (bp)
Insertion
Non-insertion
Deletions
Sequences % (no.)
100.0% (31840)
80.0% (25472)
60.0% (19104)
40.0% (12736)
20.0% (6368)
0.0% (0)
−15
−10
−5
0
Size (bp)
Deletion
Non-deletion
Substitutions
Sequences % (no.)
55.0% (17500)
44.0% (14000)
33.0% (10500)
22.0% (7000)
11.0% (3500)
0.0% (0)
0
5
10
15
Positions substituted (number)
Substitution
Non-substitution
